# Genome-wide identification and oxacillinase OXA distribution characteristics of *Acinetobacter* spp. based on a global database

**DOI:** 10.3389/fmicb.2023.1174200

**Published:** 2023-06-01

**Authors:** Jia Li, Yang Li, Xiaoli Cao, Jie Zheng, Yan Zhang, Hui Xie, Chuchu Li, Chang Liu, Han Shen

**Affiliations:** ^1^Department of Laboratory Medicine, Nanjing Drum Tower Hospital, The Affiliated Hospital of Nanjing University Medical School, Nanjing, Jiangsu, China; ^2^Department of Nosocomial Infection Control, Nanjing Drum Tower Hospital, The Affiliated Hospital of Nanjing University Medical School, Nanjing, Jiangsu, China; ^3^Department of Acute Infectious Disease Control and Prevention, Jiangsu Provincial Center for Disease Control and Prevention, Nanjing, China

**Keywords:** *Acinetobacter* spp., *Acinetobacter baumannii*, OXA-23, OXA-66, ST2

## Abstract

**Objective:**

To use genomic analysis to identify *Acinetobacter* spp. and to explore the distribution characteristics of ß-lactamase oxallicinases (*bla*OXA) among *Acinetobacter* species globally.

**Methods:**

Genomes of global *Acinetobacter* spp. were downloaded from GenBank using Aspera batch. After quality check using CheckM and QUAST software, the genomes were annotated using Prokka software to investigate the distribution of *bla*OXAs across *Acinetobacter* spp.; a phylogenetic tree was constructed to explore the evolutionary relationship among the *bla*OXA genes in *Acinetobacter* spp. Average-nucleotide identification (ANI) was performed to re-type the *Acinetobacter* spp. BLASTN comparison analysis was implemented to determine the sequence type (ST) of *Acinetobacter baumannii* strain.

**Results:**

A total of 7,853 genomes were downloaded, of which only 6,639 were left for further analysis after quality check. Among them, 282 *bla*OXA variants were identified from the genomes of 5,893 *Acinetobacter* spp.; *bla*OXA-23 (*n* = 3,168, 53.8%) and *bla*OXA-66 (2,630, 44.6%) were the most frequent *bla*OXAs, accounting for 52.6% (3,489/6639), and the co-carriage of *bla*OXA-23 and *bla*OXA-66 was seen in 2223 (37.7%) strains. The 282 *bla*OXA variants were divided into 27 clusters according to the phylogenetic tree. The biggest clade was *bla*OXA-51-family carbapenem-hydrolyzing enzymes composed of 108 *bla*OXA variants. Overall, 4,923 *A*. *baumannii* were identified out of the 6,639 *Acinetobacter* spp. strains and 291 distinct STs were identified among the 4,904 *bla*OXA-carrying *A*. *baumannii*. The most prevalent ST was ST2 (*n* = 3,023, 61.6%) followed by ST1 (*n* = 228, 4.6%).

**Conclusion:**

OXA-like carbapenemases were the main *bla*OXA-type β-lactamase spread widely across *Acinetobacter* spp. Both *bla*OXA-23 and *bla*OXA-66 were the predominant *bla*OXAs, among all *A*. *baumannii* strains, with ST2 (belonging to CC2) being the main clone disseminated globally.

## 1. Introduction

*Acinetobacter* spp. is one of the most frequent non-fermentative gram-negative coccobacilli that is widely distributed among humans as well as in the external environment. It predominantly colonizes and infects hospitalized patients, with a variety of nosocomial infections implicated (Bergogne-Bérézin and Towner, 1996). High rates of antibiotic resistance in *Acinetobacter* spp. have been documented in numerous reports, with strains possessing OXA-type carbapenem-hydrolyzing β-lactamases (CHβLs) being particularly of concern (Lee et al., 2016). Infections caused by such strains are often extremely difficult to eradicate due to their widespread resistance to the major groups of antimicrobial agents.

Currently, *Acinetobacter* spp. has been assigned into 144 different *Acinetobacter* species, including 68 with species names and 76 unnamed taxa., with *A*. *baumannii*, *A*. *nosocomialis*, and *A*. *pittii* being the most frequent ones in healthcare setting (Lupo et al., 2018; Qin et al., 2021). With the rapid development of matrix-associated laser desorption ionization–time of flight mass spectrometry (MALDI-TOF MS) and whole genome sequencing (WGS) technology, the current classification of *Acinetobacter* spp. urgently needs to be updated, since different species have been reported to cause different infections and show different drug resistance characteristics (Antunes et al., 2014; Zajmi et al., 2022; Longo et al., 2023).

Till date, *A*. *baumannii* has been found to be of the greatest clinical importance among *Acinetobacter* species, owing to its association with a variety of nosocomial infections, including pneumonia, bacteremia, urinary tract infection, and secondary meningitis (Lee et al., 2017). Specifically, ventilator-associated pneumonia caused by such strains in intensive care units (ICUs) is of great concern (Harding et al., 2018); even more worrying is the extremely and rapidly developed drug-resistance of the strains, with extensive drug-resistance and pan-drug-resistance of *A*. *baumannii* being a public health threat (Magiorakos et al., 2012). Moreover, such strains possess the capacity of long-term survival, resulting in enhanced opportunities of transmission across patients (Antunes et al., 2014).

Multiple investigations have shown that diverse OXA β-lactamases, especially the carbapenem-hydrolyzing oxacillinase (Tooke et al., 2019), play quite important roles in the resistance of *A*. *baumannii* (Kengkla et al., 2018). In particular, *bla*OXA-23, *bla*OXA-24, *bla*OXA-51, and *bla*OXA-58 have been found to be primarily associated with carbapenem resistance in *A*. *baumannii*, with *bla*OXA-23 being the most widespread gene in most countries (Khurshid et al., 2020; Wang et al., 2021), and *bla*OXA-24 and *bla*OXA-58 being the dominant genes in specific regions (Salehi et al., 2019). *bla*OXA-51 is a chromosomally encoded β-lactamase that has been demonstrated to be universally present in all *A*. *baumannii* strains, and resistance to carbapenems have been reported when the genetic environment around the gene promoted the expression of *bla*OXA-51 gene (Takebayashi et al., 2021). Furthermore, global clone groups 1 (ST1) and 2 (ST2) have been the two major clonal groups of carbapenem resistance spreading globally (Douraghi et al., 2020; Palmieri et al., 2020).

In recent years, the number of *bla*OXA variants have been continuously increasing. Correspondingly, the grouping of *bla*OXA enzymes has also changed based on amino acid sequence similarity (Walther-Rasmussen and Høiby, 2006). Till date, more than multiple *bla*OXA variants have been identified (Boyd et al., 2022; https://www.ncbi.nlm.nih.gov/gene/?term=OXA). However, the prevalence and evolution of *bla*OXAs among *Acinetobacter* spp. have remained unknown as the major β-lactamase, and whether the dissemination of *bla*OXA genes is related to specific clones would require further exploration.

In this study, we aimed to analyze the prevalent distribution of *bla*OXAs among global *Acinetobacter* spp. from all publicly available genome sequences. A phylogenetic tree of *bla*OXA variants was constructed to explore the evolutionary relationship among them. Furthermore, all the *Acinetobacter* spp. analyzed were identified by average nucleotide identity (ANI) comparison, and sequence types (STs) of the *bla*OXA-carrying *A*. *baumannii* were explored to investigate the relationship between prevalent *bla*OXAs and predominant STs.

## 2. Materials and methods

### 2.1. Download of *Acinetobacter* spp. genomes

A total of 7,853 *Acinetobacter* spp. genomes were downloaded from NCBI genome database^1^ using Aspera, on December 21, 2021. The genomic quality of the 7,853 strains was filtered by CheckM and QUAST software (Gurevich et al., 2013; Parks et al., 2015). The conditions for being considered a high-quality genome included completeness ≥ 90% and contamination < 5%. The quantity of contig was required to be ≤500 bp and N50 ≥ 40,000 bp; thus, 1,214 genomes that did not meet the above conditions were filtered out.

### 2.2. Investigation of *bla*OXAs across *Acinetobacter* spp.

Prokka (Seemann, 2014) is a fast prokaryotic genome annotation software that was used to annotate the genomes of all 6,639 strains in our study, in order to avoid the differences in genome gene prediction by different annotation methods. Distributions of blaOXA for each genome were determined by Blast analysis using a self-building blaOXA database retrieved from the National Database of Antibiotic Resistant Organisms.^2^ Genomes harboring blaOXA were selected for further analysis.

### 2.3. Phylogenetic tree of *bla*OXA variants within *Acinetobacter* spp.

MUSCLE version 3.8.31 (Edgar, 2004) was used for nucleotide sequence alignment of 282 *bla*OXA genes. Then, the generated multiple sequence alignment file was used to build a maximum likelihood (ML) phylogenetic tree by RAxML version 8.2.11 (Alexandros and Stamatakis, 2014), with Bootstrap being set as 500 and M (model setting) being selected as “GTRCAT.” Finally, this tree was visualized with iTOL software (Letunic and Bork, 2019).

### 2.4. Species identification of *Acinetobacter* spp.

Average-nucleotide identification was performed for the genomes of all 6,639 strains (Jain et al., 2018; Supplementary Table S1), and 95% was set as the cutoff value for species identification. Briefly, the genome sequences of type strains for *Acinetobacter* were obtained based on the NCBI type strain list. FastANI version 1.3 (Jain et al., 2018) was used to calculate the ANI values with all 6,639 genomes were as query, and all genomes of type strains belonging to this genus as reference. When the ANI value between a query genome and the type strain genome was greater than or equal to 95%, this query genome were given the same species name with the type strain. When the ANI value was less than 95%, this query genome was treated as *Acinetobacter* spp.

### 2.5. Analysis of the sequence types of *Acinetobacter baumannii*

A self-made Perl program was used to extract the nucleotide coding sequence of the gene from each *A*. *baumannii* genome sequence file (GBK format). Concurrently, seven allele sequences sequences and the MLST (Pasteur) profiles of *A*. *baumannii* were downloaded.^3^ The sequence type (ST) for each genome was determined as follows: (1) all genes for each genome were searched against the housekeeping gene sequences via BLASTN, and the blast results were filtered with stringent criteria (*E*-value = 1e^−5^, identity = 100%, and coverage = 100%) to obtain the seven conserved gene-type profiles; (2) this conserved gene-type profiles in each genome were compared with the MLST profiles to determine the ST for each genome.

### 2.6. Strain meta information acquisition

Strain meta information including isolation country, and date, host, and source, etc. was extracted from the downloaded gbk file in batches using perl script. Species identification, OXA distribution as well as STs were integrated in an excel for the further analysis.

### 2.7. Statistical analysis

SPSS software was used for statistical analysis, and chi-square test was used to compare the difference in distribution of *bla*OXA between *A*. *baumannii* and non-*A*. *baumannii Acinetobacter* spp.; *p* < 0.05 was taken as significant.

## 3. Results

### 3.1. Distribution of *bla*OXA across global *Acinetobacter* spp.

**Figure 1:**
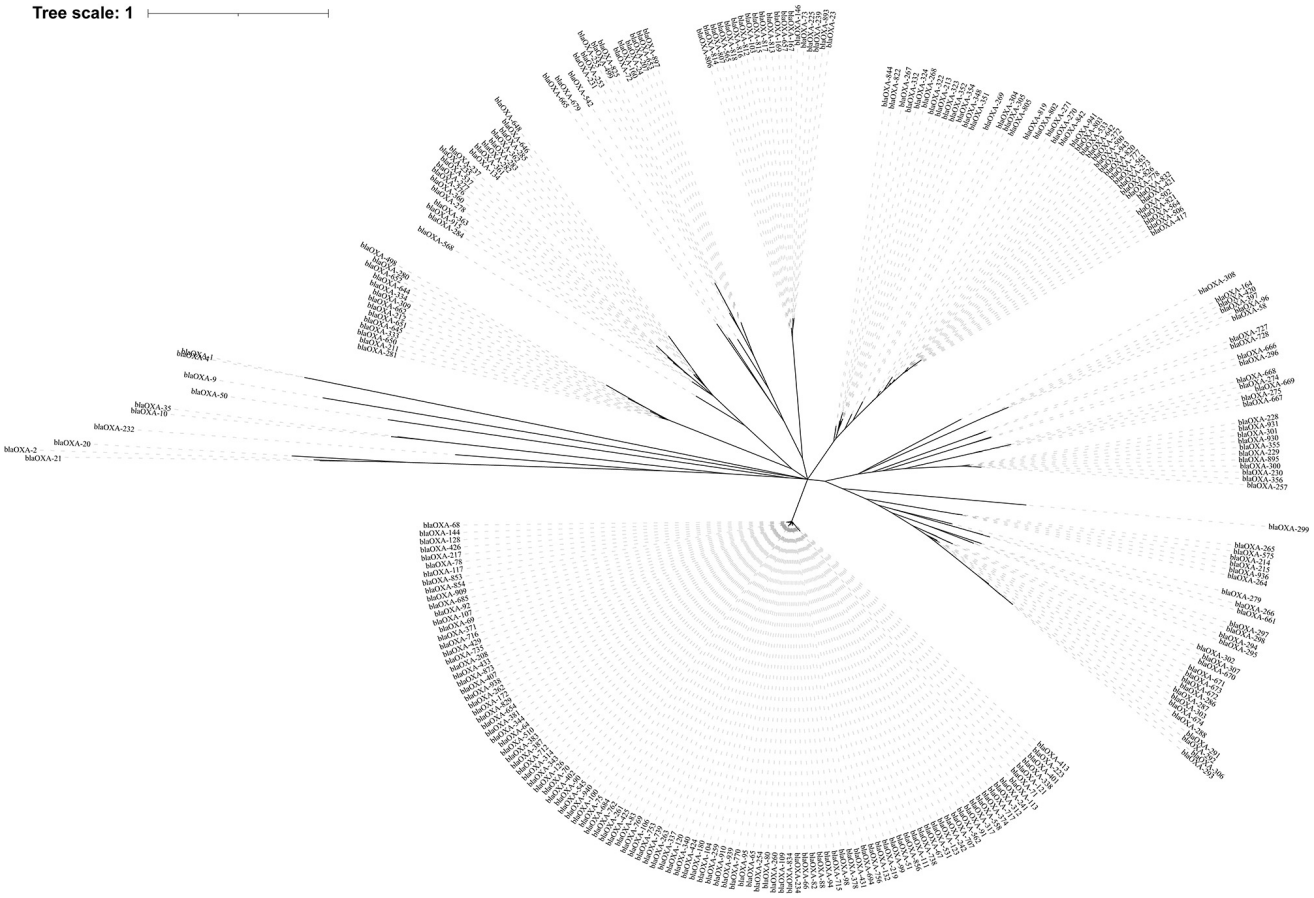
The phylogenetic tree of 282 *bla*OXA variants detected across global *Acinetobacter* spp. The phylogenetic tree was constructed using MUSCLE and RAxML software, and finally visualized with iTOL software. The clades were calculated based on the function of OXA variants.

**Figure 2:**
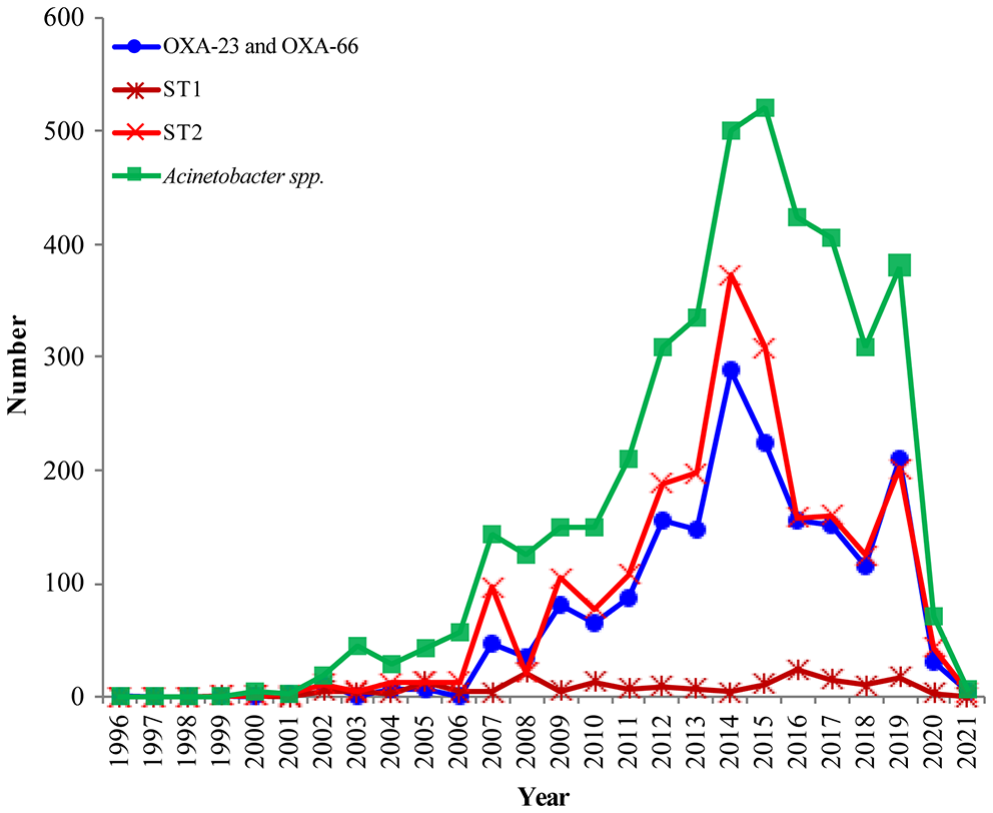
The prevalence of OXA-23 and OXA-66 combination in global *Acinetobacter baumannii* (The blue line); The distribution of OXA-producing *Acinetobacter baumannii* ST1 and ST2 during 1999–2021 (The dark red line and light red line); Analysis of the submitted and released genomes of *Acinetobacter* spp. per year (The green line).

Out of the 6,639 *Acinetobacter* spp., 5,893 (88.8%) strains were found to carry 9,581 *bla*OXAs, which were assigned to 282 *bla*OXA variants (Figure 1), with *bla*OXA-23 (*n* = 3,168, 47.7%) and *bla*OXA-66 (*n* = 2,630, 39.6%) being the most predominant *bla*OXAs. In addition, *bla*OXA-82 (*n* = 571, 8.6%), *bla*OXA-69 (*n* = 302, 4.5%), *bla*OXA-58 (*n* = 202, 3.0%), *bla*OXA-72 (*n* = 192, 2.9%), *bla*OXA-64 (*n* = 175, 2.6%), and *bla*OXA-65 (*n* = 169, 2.5%) were found to be common. Other *bla*OXAs were scattered, as shown in the Figure 1. Globally, the earliest enzyme identified was *bla*OXA-78 in 1943 in the United States. In 1980s, *bla*OXA-64 and *bla*OXA-69 were the main OXA variants, whereas *bla*OXA-214, *bla*OXA-235, *bla*OXA-500, and *bla*OXA-506 emerged successively in 1990s. Each *Acinetobacter* spp. seemed to have contained only one *bla*OXA before 1996, and subsequently co-existence of two *bla*OXA variants appeared. Since the identification of the first combination of *bla*OXA-23 and *bla*OXA-66 in *A*. *baumannii* in Singapore in 1996, it has been increasingly prevalent each year (Figure 2; Supplementary Table S2). The combinations of *bla*OXA variants detected were diverse, and distributed all over the world; a combination of three distinct *bla*OXA variants appeared in the cerebrospinal fluid of an inpatient in Italy in 2005.

Functionally, the 282 *bla*OXA variants were assigned to three classes, 239 being carbapenem-hydrolyzing-class-D-β-lactamase (Table 1), and nine belonging to oxacillin-hydrolyzing-class-D-β-lactamase (Table 2); whether the remaining 34 *bla*OXA variants possessed carbapenem-hydrolyzing activity would require further analysis (Table 3).

**Table 1:** The carbapenem-hydrolyzing-class-D-β-lactamase with *Acinetobacter spp*.

Enzyme group	Number	Enzyme (s)	Host species
OXA-23-family	20	OXA-23, OXA-73, OXA-103, OXA-146, OXA-167, OXA-169, OXA-225, OXA-239, OXA-565, OXA-657, OXA-806, OXA-807, OXA-812, OXA-813, OXA-814, OXA-815, OXA-816, OXA-817, OXA-818, and OXA-893	*A*. *baumannii*, *A*. *seifertii*, *A*. *cumulans*, *A*. *wuhouensis*, *A*. *nosocomialis*, *A*. *indicus*, *A*. *radioresistens*, *A*. *pittii*, *A*. *wuhouensis*, *A*. *cumulans*, *A*. *johnsonii*, *A*. *seifertii*, and *A*. *gandensis*
OXA-24-family	6	OXA-24, OXA-72, OXA-160, OXA-207, OXA-653, and OXA-897	*A*. *baumannii, A*. *pittii*, and *A*. *wuhouensis*
OXA-48-family	1	OXA-232	*A*. *baumannii*
OXA-51-family	108	OXA-51, OXA-64, OXA-65, OXA-71, OXA-75, OXA-78, OXA-80, OXA-82, OXA-83, OXA-88, OXA-90, OXA-92, OXA-94, OXA-95, OXA-98, OXA-99, OXA-100, OXA-104, OXA-106, OXA-107, OXA-109, OXA-111, OXA-113, OXA-117, OXA-120, OXA-121, OXA-123, OXA-126, OXA-128, OXA-132, OXA-144, OXA-172, OXA-180, OXA-208, OXA-217, OXA-219, OXA-223, OXA-241, OXA-242, OXA-254, OXA-259, OXA-260, OXA-263, OXA-312, OXA-314, OXA-317, OXA-337, OXA-338, OXA-340, OXA-343, OXA-344, OXA-371, OXA-374, OXA-378, OXA-381, OXA-383, OXA-387, OXA-401, OXA-402, OXA-407, OXA-413, OXA-424, OXA-426, OXA-429, OXA-431, OXA-433, OXA-510, OXA-531, OXA-545, OXA-558, OXA-562, OXA-654, OXA-684, OXA-685, OXA-694, OXA-707, OXA-712, OXA-715, OXA-717, OXA-735, OXA-738, OXA-739, OXA-753, OXA-756, OXA-762, OXA-769, OXA-770, OXA-829, OXA-834, OXA-853, OXA-854, OXA-856, OXA-873, OXA-909, OXA-910, OXA-938, OXA-939, and OXA-940	*A*. *baumannii*, *A*. *johnsonii*
OXA-58-family	5	OXA-58, OXA-96, OXA-164, OXA-397, and OXA-420	*A*. *baumannii*, *A*. *colistiniresistens*, *A*. *johnsonii*, *A*. *cumulans*, *A*. *seifertii*, *A*. *wuhouensis*, *A*. *bereziniae*, *A*. *haemolyticus*, *A*. *rongchengensis*, *A*. *towneri*, *A*. *lwoffii*, *A*. *pittii*, *A*. *sichuanensis*, *A*. *defluvii*, *A*. *chinensis*, *A*. *variabilis*, *A*. *nosocomialis*, *A*. *modestus*, *A*. *junii*, *A*. *indicus*, *A*. *chengduensis*, and *A*. *tianfuensis*
OXA-134-family	18	OXA-134, OXA-235, OXA-237, OXA-276, OXA-278, OXA-282, OXA-285, OXA-360, OXA-363, OXA-537, OXA-646, OXA-648, and OXA-915	*A*. *lwoffii*, *A*. *baumannii*, *A*. *schindleri*, *Acinetobacter spp*., *A*. *lwoffii*, and *A*. *pseudolwoffii*
OXA-143-family	5	OXA-231, OXA-253, OXA-255, OXA-499, and OXA-825	*A*. *baumannii*, *A*. *pittii*
OXA-211-family	14	OXA-211, OXA-212, OXA-280, OXA-281, OXA-309, OXA-333, OXA-334, OXA-498, OXA-644, OXA-645, OXA-650, OXA-652, and OXA-662	*A*. *johnsonii*, *A*. *towneri*, *A*. *cumulans*, and *Acinetobacter spp*.
OXA-213-family	42	OXA-213, OXA-267, OXA-273, OXA-304, OXA-305, OXA-322, OXA-324, OXA-332, OXA-348, OXA-351, OXA-352, OXA-354, OXA-417, OXA-421, OXA-500, OXA-502, OXA-506, OXA-533, OXA-563, OXA-564, OXA-642, OXA-777, OXA-778, OXA-802, OXA-803, OXA-805, OXA-819, OXA-822, OXA-826, OXA-832, OXA-842, OXA-844, OXA-941, and OXA-943	*A*. *calcoaceticus*, *Acinetobacter spp*., *A*. *lactucae*, *A*. *pittii*, *A*. *oleivorans*, *A*. *geminorum*, and *A*. *vivianii*,
OXA-214-family	6	OXA-214, OXA-215, OXA-264, OXA-265, OXA-575, and OXA-936	*A*. *haemolyticus*
OXA-229-family	11	OXA-228, OXA-230, OXA-257, OXA-300, OXA-301, OXA-355, OXA-356, OXA-895, OXA-930, and OXA-931	*A*. *bereziniae*, *A*. *bereziniae*, *A*. *shaoyimingii*, *A*. *piscicola*, *A*. *wuhouensis*, *A*. *rongchengensis*, and *Acinetobacter spp*.
Other carbapenem-hydrolyzing-class-D-β-lactamase	3	OXA-542, OXA-665, and OXA-666	*A*. *oleivorans*, *A*. *pittii*, *A*. *rudis*, *A*. *albensis*, *A*. *pullicarnis*, *A*. *terrestris*, and *Acinetobacter spp*.

**Table 2:** The oxacillin-hydrolyzing class D-β-lactamase in *Acinetobacter spp*.

Enzyme groups	Number	Enzyme(s)	*Acinetobacter* species
OXA-1-family	2	OXA-1, OXA-4	*A*. *colistiniresistens*, *A*. *venetianus*
OXA-2-family	2	OXA-2, OXA-21	*A*. *baumannii*, *A*. *nosocomialis*, and *A*. *pittii*
OXA-10-family	2	OXA-10, OXA-35	*A*. *pittii*, *A*. *baumannii*, and *A*. *nosocomialis*
Other oxacillinase	3	OXA-9, OXA-20, and OXA-50	*A*. *baumannii*, *Acinetobacter spp*.

**Table 3:** Class-D-β-lactamase could not be assigned into detailed classification in *Acinetobacter* spp.

Enzyme groups	Number	Enzyme(s)	*Acinetobacter* species
OXA-266-family	2	OXA-266, OXA-661	*A*. *venetianus*
OXA-274-family	5	OXA-274, OXA-275, OXA-667, and OXA-669	*A*. *guillouiae, A*. *kanungonis*, *A*. *guillouiae*, *A*. *stercoris*, *Acinetobacter spp*., and *A*. *tandoii*
OXA-286-family	15	OXA-286, OXA-288, OXA-291, OXA-293, OXA-302, OXA-303, OXA-306, OXA-307, OXA-670, and OXA-674	*A*. *proteolyticus*, *A*. *colistiniresistens*, and *Acinetobacter spp*.
OXA-667-family	1	OXA-679	*A*. *courvalinii*, *Acinetobacter spp*.
OXA-727-family	2	OXA-727, OXA-728	*A*. *chinensis*, *A*. *defluvii*, *A*. *gandensis*, and *Acinetobacter spp*.
Others	9	OXA-279, OXA-294, OXA-299, OXA-308, and OXA-568	*A*. *halotolerans*, *A*. *parvus*, *Acinetobacter spp*., *A*. *populi*, *A*. *vivianii*, *A*. *albensis*, *A*. *bohemicus*, *A*. *courvalinii*, *A*. *pragensis*, *A*. *bouvetii*, and *A*. *gerneri*

### 3.2. Phylogenetic tree of *bla*OXA variants across *Acinetobacter* spp.

Phylogenetically, the *bla*OXA variants within *A*. *baumannii* were divided into 27 distantly related clusters, according to the phylogenetic tree constructed based on SNPs (Figure 1). Among the 27 clades, 11 (including 239 *bla*OXA variants) belonged to carbapenem-hydrolyzing-class-D-β-lactamase (Figure 1; Table 1). Six clades, including nine *bla*OXA variants, belonged to oxacillin-hydrolyzing-class-D-β-lactamase (Figure 1; Table 2), and 10, including 34 *bla*OXA variants, were unknown with regard to antibiotic-hydrolyzing activity (Figure 1; Table 3). It was noteworthy that seven clades consisted of single *bla*OXA gene, namely *bla*OXA-9, *bla*OXA-50, *bla*OXA-232, *bla*OXA-568, *bla*OXA-542, *bla*OXA-308, and *bla*OXA-299. Moreover, two clades were unnamed, one group consisting of *bla*OXA-296 and *bla*OXA-666, and another group consisting of *bla*OXA-294, *bla*OXA-295, *bla*OXA-297, and *bla*OXA-298 (Table 2).

### 3.3. Species assignment of *Acinetobacter* spp.

Average-nucleotide identification calculations based on BLAST+ (ANIb) analysis (Richter et al., 2016) showed that 6,417 out of 6,639 *Acinetobacter* spp. belonged to 70 species while the remaining 222 strains could not be identified due to limitation in typing strains (Supplementary Table S1). Furthermore, the 5,893 *bla*OXA-carrying *Acinetobacter* spp. were assigned to 52 species, indicating that *bla*OXA was not identified in at least 18 *Acinetobacter* species that included *A*. *baretiae* (*n* = 2), *A*. *baylyi* (*n* = 11), *A*. *brisouii* (*n* = 4), *A*. *celticus* (*n* = 1), *A*. *equi* (*n* = 1), *A*. *guerrae* (*n* = 3), *A*. *harbinensis* (*n* = 2), *A*. *lanii* (*n* = 2), *A*. *larvae* (*n* = 1), *A*. *nectaris* (*n* = 1), *A*. *pollinis* (*n* = 4), *A*. *rathckeae* (*n* = 1), *A*. *soli* (*n* = 35), *A*. *terrae* (*n* = 8), *A*. *terrestris* (*n* = 5), *A*. *tjernbergiae* (*n* = 2), *A*. *ursingii* (*n* = 56), and *A*. *wanghuae* (*n* = 2). Further, we observed that the distribution of *bla*OXAs across *Acinetobacter* species was different, the most common species being *A*. *baumannii* (*n* = 4,904) followed by *A*. *pittii* (*n* = 299), which was then followed by *A*. *bereziniae* (*n* = 47), *A*. *haemolyticus* (*n* = 39), *A*. *johnsonii* (*n* = 40), *A*. *lwoffii* (*n* = 37), *A*. *oleivorans* (*n* = 34), and *A*. *radioresistens* (*n* = 43; Table 4). Other species were quite rare, as shown in Supplementary Table S1. Notably, 174 *bla*OXA-carrying *Acinetobacter* spp. could not be assigned to specific species due to the limited type strains in GenBank.

**Table 4:** The distribution of major OXA-variants among predominant *Acinetobacter* spp.

Strains	Number of OXA-carrying Strains (%)	Number of OXA-variants	The most prevalent OXA-variants		
*A*. *baumannii*	4,904/4,923 (99.6%)	136	OXA-23	OXA-66	OXA-82
			(*n* = 3,129)	(*n* = 2,621)	(*n* = 790)
*A*. *pittii*	299/305 (98.0%)	36	OXA-500	OXA-506	OXA-421
			(*n* = 146)	(*n* = 27)	(*n* = 25)
*A*. *bereziniae*	47/47 (100%)	11	OXA-355	OXA-301	OXA-301/356
			(*n* = 14)	(*n* = 11)	(*n* = 4)
*A*. *johnsonii*	40/42 (95.2%)	20	OXA-281	OXA-644	OXA-58
			(*n* = 7)	(*n* = 7)	(*n* = 6)
*A*. *haemolyticus*	39/39 (100%)	7	OXA-264	OXA-214	OXA-265
			(*n* = 13)	(*n* = 11)	(*n* = 8)
*A*. *lwoffii*	37/40 (92.5%)	12	OXA-283	OXA-282	OXA-362
			(*n* = 15)	(*n* = 7)	(*n* = 3)
*A*. *oleivorans*	34/35 (97.1%)	4	OXA-304	OXA-305	OXA-805
			(*n* = 19)	(*n* = 13)	(*n* = 2)
*A*. *indicus*	23/130 (17.7%)	4	OXA-58	OXA-146	OXA-23
			(*n* = 14)	(*n* = 6)	(*n* = 2)
*A*. *calcoaceticus*	22/22 (100%)	10	OXA-332	OXA-213	OXA-268/351
			(*n* = 8)	(*n* = 4)	(*n* = 2)
*A*. *nosocomialis*	16/217 (7.4%)	7	OXA-58	OXA-96	OXA-23
			(*n* = 7)	(*n* = 3)	(*n* = 3)
*A*. *radioresistens*	43/44 (97.7%)	11	OXA-23	OXA-816	OXA-813
			(*n* = 10)	(*n* = 7)	(*n* = 5)
*A*. *schindleri*	21/21 (100%)	8	OXA-360	OXA-537	OXA-276/277
			(*n* = 7)	(*n* = 3)	(*n* = 3)
*Acinetobacter spp*.	174/222 (78.4%)	46	OXA-728	OXA-58	OXA-727
			(*n* = 54)	(*n* = 23)	(*n* = 19)

It would be worth noting that the most prevalent *bla*OXA variants were different across the *Acinetobacter* species (Table 4). For example, *bla*OXA-23 and *bla*OXA-66 were the predominant *bla*OXA variants in *A*. *baumannii*, whereas *bla*OXA-500 and *bla*OXA-506 were the dominant ones in *A*. *pittii*. Of note, more than two *bla*OXA variants were identified across 3,615 strains, and 2,223 were found to simultaneously carry *bla*OXA-23 and *bla*OXA-66; co-carriage of *bla*OXA-23 and *bla*OXA-66 was only found in *A*. *baumannii*. The other difference was that *bla*OXA-66 was only identified in *A*. *baumannii*, whereas *bla*OXA-23 was not only detected in *A*. *baumannii* (*n* = 3,129), but also in *A*. *cumulans* (*n* = 2), *A*. *gandensis* (*n* = 2), *A*. *indicus* (*n* = 2), *A*. *johnsonii* (*n* = 1), *A*. *nosocomialis* (*n* = 4), *A*. *pittii* (*n* = 6), *A*. *radioresistens* (*n* = 10), *A*. *seifertii* (*n* = 3), and *A*. *wuhouensis* (*n* = 1).

Albeit there was a wide distribution of *bla*OXAs across *A*. *bereziniae*, *A*. *haemolyticus*, *A*. *johnsonii*, *A*. *lwoffii*, *A*. *oleivorans*, *A*. *pittii*, *A*. *radioresistens*, and *A*. *baumannii* (Table 4), in general, the prevalence of *bla*OXAs among *A*. *baumannii* (4,904/4,923, 99.6%) was significantly higher than that of *bla*OXAs among *non-A*. *baumannii Acinetobacter* spp. (989/1716, 57.6%, *p* = 0.000).

The distribution of carbapenem-hydrolyzing-class-D-β-lactamases showed that *bla*OXA-51 family, the biggest clade, was only found in *A*. *baumannii* and *A*. *johnsonii*. Other carbapenemases, including *bla*OXA-134, *bla*OXA-211, *bla*OXA-213, *bla*OXA-214, and *bla*OXA-229 families, as well as other enzymes not assigned into a specific family were not found in *A*. *baumannii*.

### 3.4. Sequence types of *bla*OXA-carrying *Acinetobacter baumannii*

**Figure 3:**
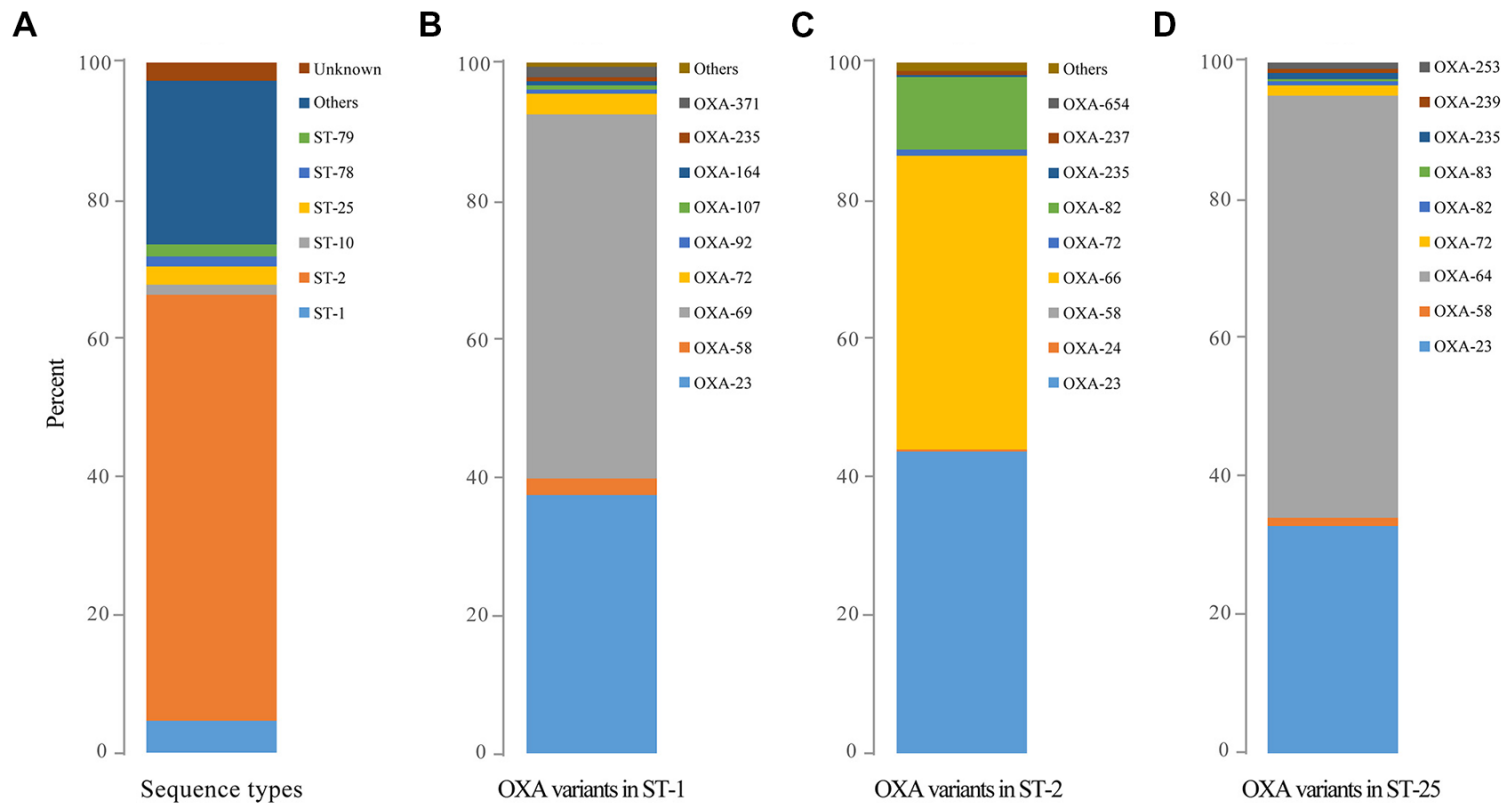
The predominant sequence types of global OXA-producing *Acinetobacter baumannii* in 1996, 1999, and during 2001–2021 as well as the distribution of OXA variants among the three dominant clones of *Acinetobacter baumannii*. **(A)** The predominant sequence types of global OXA-producing *Acinetobacter baumannii* in 1996, 1999, and during 2001–2021; **(B)** The distribution of OXA variants among *Acinetobacter baumannii* ST1; **(C)** The distribution of OXA variants among *Acinetobacter baumannii* ST2; and **(D)** The distribution of OXA variants among *Acinetobacter baumannii* ST25.

A total of 291 distinct STs were identified for 4,904 *bla*OXA-carrying *A*. *baumannii*. The most identified ST was ST2 (*n* = 3,023), which was classified as clonal complex 2 (CC2), presenting a global distribution trend. ST1 (*n* = 228; clonal complex CC1) and ST25 (*n* = 132), which were the second most common, corresponded to CC2/92 (Pasteur/Oxford scheme; Figure 3A), other distinct STs (ST79, ST78, and ST10) were relatively less common (Figure 3A). The STs of 29 strains were novel (the profile of MLST gene was not assigned into specific ST), and the STs of 125 strains remained unknown (the STs could not be determined since some genes did not match the known MLST loci).

The earliest detected clone in *A*. *baumannii* was ST2 collected in Rotterdam, Netherlands, in 1982 from *Homo sapiens*, which was further found in 1996 in Singapore. The clonal ST2 was continuously and increasingly detected every year all over the world since its first detection in Beijing, China, in 1999 (Figure 2, The light red line). Additionally, ST1 was consecutively collected almost each year, except in 1985, 1995, and 2001 (Figure 2, The dark red line); however, the number was quite less compared to that of ST2 (Figure 2, The light red line).

Ninety-two distinct STs were identified for *bla*OXA-23-carrying strains, with ST2 (*n* = 2,429) being the most prevalent clone, followed by ST1 (*n* = 154). Meanwhile, 25 different STs were found for 2,663 *bla*OXA-66-carrying *A*. *baumannii* with ST2 (*n* = 2,425) being the most common. Notably, 2081 *bla*OXA-23 and *bla*OXA-66 co-carrying *A*. *baumannii* belonged to ST2 while the other 22 STs were also identified.

Obvious differences were also observed among the major STs. *bla*OXA-23 (*n* = 2,429) and *bla*OXA-66 (*n* = 2,374) were the main *bla*OXAs within ST2, whereas *bla*OXA-69 (*n* = 217) and *bla*OXA-23 (*n* = 154) were the predominant *bla*OXAs among ST1, and *bla*OXA-64 (*n* = 128) was the dominant *bla*OXA among ST25 clones (Figures 3B–D).

### 3.5. Temporal and geographical distribution of global *bla*OXA-carrying *Acinetobacter* spp.

**Figure 4:**
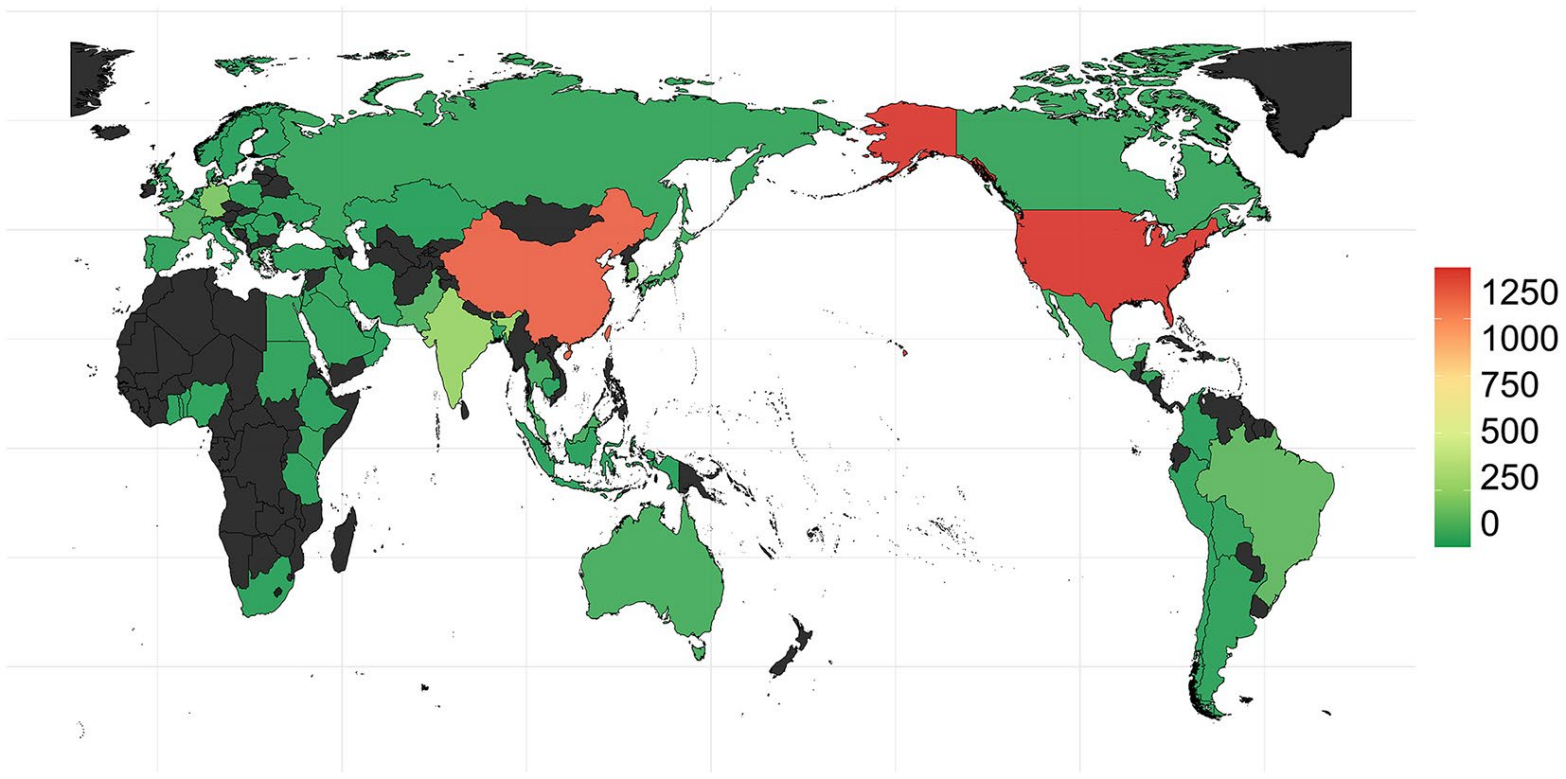
Submitted and released genomes of *Acinetobacter* spp. by different countries worldwide.

The earliest OXA-producing *Acinetobacter* spp. could be dated back to 1943, and was carried by an *A*. *baumannii* from *Parthenium argentatum* Gray (guayule shrubs) in the United States. The *bla*OXA-producing *Acinetobacter* spp. was intermittently isolated over the following years: 1948 (*n* = 2), 1951 (*n* = 1), 1953 (*n* = 1), 1982 (*n* = 2), 1984 (*n* = 1), 1985 (*n* = 1), and 1986 (*n* = 1), and was found to continuously increase during 1991 to 2021, although the isolation date for 1,585 strains still remains unknown (Figure 2). Overall, the strains were collected from 79 countries (Figure 2; Supplementary Table S3), of which, United States (*n* = 1,315) and China (*n* = 1,191) submitted the most number of strains, followed by Germany (*n* = 205), India (*n* = 279), South Korea (*n* = 145), Brazil (*n* = 134), and France (*n* = 101; Figure 4; Supplementary Table S3). Unfortunately, the countries from where the other 1,345 strains were collected remain unknown. Furthermore, *Homo sapiens* were the most common host. The *Acinetobacter* spp. isolated only from non-*Homo sapiens* were *A*. *chengduensis*, *A*. *chinensis*, *A*. *cumulans*, *A*. *gandensis*, *A*. *kanungonis*, *A*. *modestus*, *A*. *piscicola*, *A*. *populi, A*. *pragensis, A*. *pseudolwoffii, A*. *rongchengensis, A*. *pullicarnis, A*. *shaoyimingii, A*. *sichuanensis, A*. *terrestris, A*. *tianfuensis, A*. *towneri, A*. *variabilis,* and *A*. *wuhouensis*.

The distribution of *bla*OXAs displayed significant regional differences. *bla*OXA-23 and *bla*OXA-66, in combination, were the main *bla*OXAs in Asian regions, such as China, South Korea, Singapore, Pakistan, Malaysia, Lebanon, Kuwait, Thailand, and India. However, *bla*OXA-23 and *bla*OXA-82 were the predominant ones in United States.

## 4. Discussion

In this study, we analyzed the data available on the distribution of *bla*OXAs across the genomes of the *Acinetobacter* spp. available in the GenBank, and the evolutionary relationship among the *bla*OXA variants based on the global genomes of *Acinetobacter* spp. Furthermore, the relationship between *bla*OXA variants and STs in *A*. *baumannii* was analyzed.

First, consistent with the previous report, we found a wide distribution of diverse OXA-type β-lactamases across the *Acinetobacter* spp., especially in *A*. *baumannii,* indicating that *A*. *baumannii* might be the main host of *bla*OXA genes. It was noteworthy that most of the OXA-type β-lactamase identified in our study belonged to carbapenem-hydrolyzing-β-lactamase, leading to high resistance of *Acinetobacter* spp. to carbapenem in different degrees (Boral et al., 2019; Tamma et al., 2022). Evidently, the wide distribution of *bla*OXA-23 and *bla*OXA-66 across global *A*. *baumannii*, in our study, suggested that they may be the main enzyme mediating carbapenem resistance, since the expression of *bla*OXA-23 within the *A*. *baumannii* strain was enough to confer resistance to carbapenems (Evans and Amyes, 2014); however, a much higher turnover rate was observed for imipenem than for meropenem, ertapenem, or doripenem for *bla*OXA-23 (Smith et al., 2013). In addition, a recent study showed that hyperexpression of *bla*OXA-23 β-Lactamase in *A*. *baumannii* drives significant collateral changes with increased amidase activity, resulting in peptidoglycan integrity and new genetic vulnerabilities (Colquhoun et al., 2021), which may represent novel targets for antimicrobial agents. *bla*OXA-66 is well known as a chromosomally encoded *bla*OXA-51-like β-lactamase, and the most prevalent CHβLs in *A*. *baumannii* worldwide (Hu et al., 2007). Therefore, the wide co-occurrence of *bla*OXA-23 and *bla*OXA-66 in combination with other diverse carbapenem-hydrolyzing enzymes within *A*. *baumannii* provides enough explanation for the failure of β-lactam in clinical therapy. Currently, many studies have shown frequent co-carriage of *bla*OXA-23 and *bla*OXA-66 among clinical *A*. *baumannii*, with high resistance rates (Al-Hassan et al., 2021; Zhang et al., 2021), and the continuously rising co-prevalence of *bla*OXA-23 and *bla*OXA-66 over the years indicates the importance of the two variants for high resistance of *A*. *baumannii* to carbapenem.

In our study, *bla*OXA-78 (OXA-51 family) was the earliest *bla*OXA in *A*. *baumannii* isolated in 1943 in United States. However, searching *bla*OXA-78 as a keyword in PubMed, it was first reported in a clinical multi-drug resistant *A*. *baumannii* from a hospitalized patient in a major hospital in Kuwait in 2015 (Vali et al., 2015). As is well known, carbapenem was approved for clinical use in 1980s, indicating that class-D-CHßLs had already existed in *A*. *baumannii* before carbapenem usage. Analysis from our study showed that *bla*OXA-51 family, including *bla*OXA-66, *bla*OXA-69, and *bla*OXA-98, appeared in bacterial genomes submitted in the 1980s, and OXA-213 family, including *bla*OXA-500, *bla*OXA-506, and *bla*OXA-417, emerged in strains collected in the 1990s, although the *bla*OXAs were first reported in 2007 (Zhou et al., 2007), and *bla*OXA-69 in 2005 (Héritier et al., 2005), *bla*OXA-417 in 2014 (D'Souza et al., 2019), and *bla*OXA-500 in 2019 (Sun et al., 2014), indicating the presence of these *bla*OXA before the use of carbapenems. Whether there was an association between the evolution of class D CHβLs and the carbapenem use would require further investigation. Importantly, all the *bla*OXA-48-like CHβLs, were quite rare, except *bla*OXA-232, which was quite popular in *Enterobacterale*s (Pitout et al., 2019). This could be due to the good fitness between *bla*OXA-48 and *Enterobacterales*, especially *Klebsiella pneumoniae*.

In our study, the clades of *bla*OXA variants were in accordance with the subgroups categorized by enzyme function, from an overall perspective, and *bla*OXA-51 family included the most members. However, not all the *A*. *baumannii* isolates contained *bla*OXA-51-family genes, providing evidence that this gene was not omnipresent in the species, but rather distributed in subpopulations of *A*. *baumannii* (Walther-Rasmussen and Høiby, 2006). Nevertheless, the members of this family were reported to diverge by amino acid modifications in *A*. *baumannii* (Brown and Amyes, 2005; Héritier et al., 2005), contributing to intrinsic resistance to imipenem (Hu et al., 2007). In addition, *bla*OXA family appears to be evolving quite quickly in recent years as supported by the presence of 282 *bla*OXA variants across all *Acinetobacter* spp. globally, along with more yet-unknown variants, since most of the recently submitted genomes had not been released when the related genomes were downloaded for analysis.

We found quite a wide distribution of ST2, most of them co-carrying *bla*OXA-23 and *bla*OXA-66, indicating an international clonal dissemination of the strains, mainly among *Homo sapiens* in health care centers in 38 countries and six continents across the world, posing a serious threat to global public health. As the clone secondary to ST2, although ST1 clone only accounted for little part of the strains, we not only found an increasing trend of ST1 clone every year, but also a wide distribution across 30 countries, mainly Brazil, United States, and Australia. Interestingly, ST1 was not found in isolates from China. The other multiple STs identified in our study indicated the diversity of *bla*OXA-carrying *A*. *baumannii* strains.

According to the current WGS identification and typing methods for *Acinetobacter* spp., at least 70 members of this genus were identified. It would be worth mentioning that the naming of at least 27 existing genomes was wrong. For example, “*A*. *colistiniresistens*” was mistaken as “*A*. *baumannii*,” indicating that most *Acinetobacter* species were misidentified previously, and the underlying reason could be that NCBI started ANI verification-taxonomy nomenclature around 2016, and there was no ANI verification for previously submitted genomes. Moreover, the genomes of typing strains submitted have been increasing, which will also affect their taxonomy verification. If there is no obvious error, the submitted genome with spp. may pass automatically.

There were some limitations in our current study. First, some strains may have been excluded from the analysis, since only the *A*. *baumannii* strains, whose genomes were submitted to GenBank and released, were analyzed, even though the analysis was based on global data. Second, the resistant profiles of these strains were missing; we could not check the accordance between the phenotype and genotypes of these strains. Third, all the results were acquired based on WGS, and the strains were not available for further confirmation by molecular methods.

In summary, class D β-lactamase *bla*OXA variants in *Acinetobacter* spp. have been rapidly evolving, with CHβLs being the most predominate class D β-lactamase, widely distributed within *Acinetobacter* spp. *bla*OXA-23- and *bla*OXA-66-co-carrying *A*. *baumannii* ST2 is a predominant international high-risk clone spreading globally that poses potential threat to global public health.

## Data availability statement

The datasets presented in this study can be found in online repositories. The names of the repository/repositories and accession number(s) can be found in the article/Supplementary material.

## Author contributions

JL, YL, and XC performed the bioinformatics analysis and writing. JZ and YZ sorted the data and helped with the writing. HX interpreted the data regarding resistance determinants and plasmid replicons. CLi performed statistical analysis. CLiu and HS designed the work and were major contributors in revising the manuscript. All authors contributed to the article and approved the submitted version.

## Funding

This work was supported by the National Natural Science Foundation of China (grant numbers 81902124 and 82002205).

## Conflict of interest

The authors declare that the research was conducted in the absence of any commercial or financial relationships that could be construed as a potential conflict of interest.

## Publisher’s note

All claims expressed in this article are solely those of the authors and do not necessarily represent those of their affiliated organizations, or those of the publisher, the editors and the reviewers. Any product that may be evaluated in this article, or claim that may be made by its manufacturer, is not guaranteed or endorsed by the publisher.

## Author disclaimer

The views expressed in this article are those of the authors and do not necessarily reflect the official policy or position of the Department of Laboratory Medicine, Nanjing Drum Tower Hospital, the Affiliated Hospital of Nanjing University Medical School, and the Clinical Research Center, the Second Hospital of Nanjing, Nanjing University of Chinese Medicine, Nanjing, China.

## Footnotes

^1^https://www.ncbi.nlm.nih.gov/genome/browse/

^2^https://www.ncbi.nlm.nih.gov/pathogens/refgene/#blaOXA

^3^https://pubmlst.org/

## Abbreviations

CHβL: Carbapenem-hydrolyzing β-lactamase
MALDI-TOF MS: Matrix-associated laser desorption ionization–time of flight mass spectrometry
WGS: Whole genome sequencing
ICU: Intensive care unit
ANI: Average nucleotide identification
ST: Sequence type
SNP: Single nucleotide polymorphism
MLST: Multi-locus sequence typing
